# Limited educational attainment and radiographic and symptomatic knee osteoarthritis: a cross-sectional analysis using data from the Johnston County (North Carolina) Osteoarthritis Project

**DOI:** 10.1186/ar2956

**Published:** 2010-03-18

**Authors:** Leigh F Callahan, Jack Shreffler, Bernadette C Siaton, Charles G Helmick, Britta Schoster, Todd A Schwartz, Jiu-Chiuan Chen, Jordan B Renner, Joanne M Jordan

**Affiliations:** 1 Thurston Arthritis Research Center, Department of Medicine, 3300 Thurston Building, CB # 7280, University of North Carolina, Chapel Hill, NC 27599-7330, USA; 2 Thurston Arthritis Research Center, Department of Orthopaedics, 3300 Thurston Building, CB # 7280, University of North Carolina, Chapel Hill, NC 27599-7330, USA; 3 Thurston Arthritis Research Center, Department of Social Medicine, 3300 Thurston Building, CB # 7280, University of North Carolina, Chapel Hill, NC 27599-7330, USA; 4 Duke University Medical Center, DUMC 3918, Durham, NC 27710, USA; 5 Centers for Disease Control and Prevention, 1600 Clifton Rd, Atlanta, GA 30333, USA; 6 Department of Biostatistics, Gillings School of Global Public Health, University of North Carolina, 3106E McGavran-Greenberg Hall, CB #7420, Chapel Hill, NC 27599-7420, USA; 7 Division of Environmental Health, Keck School of Medicine of the University of Southern California, 1975 Zonal Avenue, KAM 500, Los Angeles, CA 90089-9034, USA; 8 Department of Radiology, University of North Carolina, 509 Old Infirmary Bldg, Campus Box 7510, Chapel Hill, NC 27599, USA

## Abstract

**Introduction:**

Applying a cross-sectional analysis to a sample of 2,627 African-American and Caucasian adults aged ≥ 45 years from the Johnston County Osteoarthritis Project, we studied the association between educational attainment and prevalence of radiographic knee osteoarthritis and symptomatic knee osteoarthritis.

**Methods:**

Age- and race-adjusted associations between education and osteoarthritis outcomes were assessed by gender-stratified logistic regression models, with additional models adjusting for body mass index, knee injury, smoking, alcohol use, and occupational factors.

**Results:**

In an analysis of all participants, low educational attainment (<12 years) was associated with higher prevalence of four knee osteoarthritis outcomes (unilateral and bilateral radiographic and symptomatic osteoarthritis). Women with low educational attainment had 50% higher odds of having radiographic knee osteoarthritis and 65% higher odds of symptomatic knee osteoarthritis compared with those with higher educational attainment (≥ 12 years), by using fully adjusted models. In the subset of postmenopausal women, these associations tended to be weaker but little affected by adjustment for hormone replacement therapy. Men with low educational attainment had 85% higher odds of having symptomatic knee osteoarthritis by using fully adjusted models, but the association with radiographic knee osteoarthritis was explained by age.

**Conclusions:**

After adjustment for known risk factors, educational attainment, as an indicator of socioeconomic status, is associated with symptomatic knee osteoarthritis in both men and women and with radiographic knee osteoarthritis in women.

## Introduction

Educational attainment has often been used as a surrogate for socioeconomic status (SES) in analyses of the role of SES in health outcomes [1] because it is unlikely to change in adults and is easily obtained in a clinical setting. Individuals from the general population with lower educational attainment have increased chronic-disease prevalence, morbidity, and mortality [2-4], as do individuals with specific diseases, such as cardiovascular disease, diabetes, hypertension, and chronic lung disease [5-9].

In the rheumatic diseases, extensive research has been conducted on the relation between formal educational level and rheumatoid arthritis (RA) [7,8,10-14], but less is known about a possible association between educational attainment and osteoarthritis (OA). Several studies have noted associations between lower educational attainment and both OA prevalence and poorer outcomes, including pain and disability [15-20]. However, only one study used radiographic OA data. In the first National Health and Nutrition Examination Survey (NHANES-I, 1971 to 1975), Hannan and colleagues [16] found that radiographic and symptomatic knee OA were both associated with low educational attainment, but only the association with symptomatic knee OA remained independent of age, knee injury, race, obesity, and occupation.

Adults with limited educational attainment are more likely to be older or African American, to smoke, to have higher body mass index (BMI), and to work in more physically demanding occupations [21,22], all risk factors for radiographic knee OA or various musculoskeletal symptoms [23-25]. Additionally, among postmenopausal women, those with limited educational attainment may lack access to potentially protective factors, such as hormone replacement therapy (HRT) [26-31], which has been inversely associated with radiographic knee OA [26,32,33].

We have extended the work of Hannan by examining associations between educational attainment and four knee OA-related outcomes (radiographic and bilateral radiographic knee OA and symptomatic and bilateral symptomatic knee OA) in a cohort of participants from the baseline (1990 to 1997) enrollment in the Johnston County Osteoarthritis Project, a community-based study of OA in African American and Caucasian men and women aged ≥ 45 years in a rural, North Carolina county [34]. We hypothesized that those with limited educational attainment would be more likely than those with higher educational attainment to have these OA outcomes and that these associations might persist even after adjustment by demographic, lifestyle, clinical, or occupational factors. Among postmenopausal women, we also hypothesized that lack of exposure to HRT might be a potential explanation for the higher odds of radiographic knee OA in those with low educational attainment.

## Materials and methods

Participants were non-Hispanic African Americans and Caucasians in the Johnston County Osteoarthritis Project, a population-based study of OA in a rural North Carolina county.

As a starting point, 3,047 participants were available for this study, a sample virtually identical to that analyzed in the previous article on prevalence of knee OA and symptomatic OA [34]. Ultimately, the sample was reduced to 2,627 participants who had complete, nonmissing values for all explanatory variables of interest. Details of the sampling strategy and protocol are delineated in the previous report [35]. This study was approved by the Institutional Review Boards of the University of North Carolina Schools of Medicine and Public Health and the Centers for Disease Control and Prevention. All participants gave written informed consent at the time of recruitment.

In brief, civilian, non-institutionalized African Americans and Caucasians 45 years of age or older who were physically and mentally capable of completing the protocol involving radiographs, interview, and physical examination were eligible. During the period from 1991 through 1997, participants were recruited by probability sampling from six townships in the county. Response rate was approximately 72%, and respondents did not vary from nonrespondents by age, sex, ethnic group, formal education level, or presence of knee pain [34]. All participants completed two home, interviewer-administered interviews, and a limited clinical and functional examination, including assessment of weight (kg) by using a balance-beam scale, height (cm) measured with a stadiometer, and radiographic examination of the knees to diagnose radiographic tibiofemoral knee OA or symptomatic tibiofemoral knee OA. The following variables enter this analysis.

### Educational attainment

Participants were asked to report the number of years of formal education that they had completed. Educational attainment was dichotomized at the completion of high school (<12 years vs. ≥ 12 years).

### Demographic characteristics

Participants were queried about their age (45 through 54, 55 through 64, 65 through 74, or ≥ 75), race (Caucasian, African American), and gender (male, female).

### Lifestyle/clinical characteristics

Participants were asked about current smoking of tobacco (yes, no), current alcohol use (yes, no), and history of previous knee injury (yes, no). Body mass index (BMI, kg/m^2^) was calculated from weight and height. For the modeling, BMI was divided by 5 and treated as a continuous variable.

### Occupational characteristics

Two variables were used to describe occupation and work-related physical demands, assessed by using both current and previous employment. First, self-reported occupations (either current or previous, if the participant was retired) were classified into six broad groups according to the codes listed in the 1990 Census of Population and Housing Alphabetical Index of Industries and Occupations: (a) Managerial and Professional Specialty Occupations, (b) Technical, Sales, and Administrative Support, (c) Service Occupations, (d) Farming, Forestry, and Fishing, (e) Precision Production, Craft, and Repair Occupations, and (f) Operators, Fabricators, and Laborers [36]. Based on underlying Census classifications, reported occupations were dichotomized as physically demanding (PD) (c-f) or not physically demanding (a and b). Second, an occupational physical activity score was created from participants' rating of the frequencies of four physical activities (squatting, standing, lifting heavy objects, and walking) at work, each assessed on a 5-point scale (never = 0; seldom = 1; sometimes = 2; often = 3; always = 4). The scores (scale 0-4) for each activity were computed as the maximum rating for either the present or previous employment and summed to create a physical activities scale (0 to 16). For modeling, the score was dichotomized as low (<10) or high (≥ 10).

### Hormone replacement therapy

Postmenopausal women were asked to describe any use of HRT (including hormone pills, shots, or implants), and the response was dichotomized as yes or no.

### Radiographic knee OA

Anteroposterior knee radiographs with weight bearing were obtained in standard format with foot map positioning and assessed by a single bone and joint radiologist (JBR) for Kellgren-Lawrence (K/L) grade by using a standard knee atlas [37]. K/L grades for the tibiofemoral joint were defined as follows: 0 = no OA; 1 = questionable OA; 2 = mild OA; 3 = moderate OA; and 4 = severe OA [35,37]. Inter- and intrarater reliabilities have been reported (weighted K = 0.87 and 0.89, respectively) [37]. Participants with films suggesting underlying inflammatory arthropathy, such as RA, were eliminated from the study. Radiographic tibiofemoral knee OA was defined as a K/L grade of ≥ 2 in at least one knee. Bilateral radiographic tibiofemoral knee OA was defined as K/L grade of ≥ 2 in both knees.

### Symptomatic knee OA

Symptomatic knee OA was defined as the presence of radiographic knee OA plus an affirmative response for that same knee, to the question, "On most days, do you have pain, aching, or stiffness in your (right, left) knee?" Bilateral symptomatic knee OA was defined as the presence of radiographic knee OA plus pain, aching, or stiffness on most days in both knees.

### Statistical analysis

A missing value in any of the variables will cause all data of the participant to be dropped from a regression analysis, and, in this sample of 3,047 participants, 420 people had at least one of the explanatory variables listed as missing, and so the effective sample for regression analysis was restricted to 2,627. The most common missing variable was Census code for occupation, most frequently in women who had not worked outside the home for >1 year. Physical activities related to occupation and information on knee injury were the next most frequently missing variables. Owing largely to missing occupational codes, women, who were 60.5% of the original sample, decreased to 59.9% of the final, restricted sample. In reality, both the characteristics of the sample and regression findings are negligibly affected by the restriction of sample size, as further discussed in Results.

The four OA outcomes (radiographic knee OA, bilateral radiographic knee OA, symptomatic knee OA, and bilateral symptomatic knee OA) are represented by dichotomous (no = 0/yes = 1) variables. The indicator for radiographic OA is set to 1 if OA is present (as previously defined by K/L grade) in one or both knees, and it is set to 0 if no OA is present in either knee. The indicator for bilateral radiographic OA is set to 1 if OA is present in both knees. It is set to zero if no OA is found in either knee, and it is set to *missing *if OA is present in one and only one knee (unilateral OA). A parallel representation applies to symptomatic OA and bilateral symptomatic OA. Because of the unilateral findings, the numbers of participants entering the bilateral analyses are less than those in the general analyses.

All analyses were performed separately for men, women, and the subgroup of postmenopausal women by using Stata Version 9 software. Unweighted descriptive statistics were tallied for the demographic, occupational, clinical, and radiographic characteristics of the restricted sample. Characteristics by educational categories were compared, as appropriate, with χ^2 ^tests or with Student's *t *tests. Within each gender category, associations between educational attainment and each of the four knee OA outcomes were modeled by a series of logistic regressions, with groups of covariates added cumulatively to the models. The first model adjusts for age and race, the second model adds adjustment for four lifestyle and clinical factors, and the third model adds adjustments for two occupational factors. The subgroup analysis of postmenopausal women [38] includes a final model additionally adjusting for HRT use. Because the sample was restricted to nonmissing explanatory variables, no participants were dropped as covariates were added.

## Results

Data from 2,627 individuals were available from the Johnston County Osteoarthritis Project, including 558 African-American women, 273 African-American men, 1,016 Caucasian women, and 780 Caucasian men. Overall, the mean age of the men was 60.6 ± 10.2 years, and the mean age of the women was 60.8 ± 10.6 years. Thirty-eight percent of men and 37% of women had low educational attainment (<12 years). Of the 1,330 postmenopausal women, 455 were African American, and 875 were Caucasian. Mean age for all women in this subset was 62.4 ± 10.1 years. Of these, 39% had low educational attainment, and 24% were current HRT users.

For the complete sample, 29% had radiographic OA, 17% had bilateral radiographic OA, 16% had symptomatic OA, and only 6.5% had bilateral symptomatic OA. Prevalences of all OA outcomes were somewhat higher for women than for men.

Those with low educational attainment were more likely to be older, African American, to work in more physically demanding occupations, to have more radiographic and symptomatic knee OA, and to be less likely to report current alcohol use (Table 1). Men with low educational attainment were more likely to smoke and have lower BMIs than were men with higher educational attainment, whereas the opposite was true for women and postmenopausal women. Postmenopausal women with low educational attainment were less likely to report use of an HRT (Table 1).

**Table 1:** Characteristics of the study sample according to gender and educational attainment

	**Men** **(n = 1,053)**			**Women** **(n = 1,574)**			**Postmenopausal Women** **(n = 1,330)**		
						
	Educational attainment								
	Low	High	*P *value	Low	High	*P *value	Low	High	*P *value
	(n = 402)	(n = 651)		(n = 587)	(n = 987)		(n = 518)	(n = 812)	
Demographic characteristics									
% Age group in years			<0.001			<0.001			<0.001
45-54	19.7	42.9		17.6	44.5		12.2	35.3	
55-64	23.9	32.1		24.2	33.2		25.9	38.4	
65-74	37.8	20.7		35.8	15.8		37.8	18.6	
75+	18.7	4.3		22.5	6.5		24.1	7.6	
% African American	36.1	19.7	<0.001	43.6	30.6	<0.001	42.9	28.7	<0.001

Lifestyle/clinical characteristics									
% Current Smokers	32.1	24.9	0.011	13.3	17.9	0.016	13.3	18.4	0.016
% Current alcohol use	20.4	34.1	<0.001	6.6	17.9	<0.001	6.2	17.2	<0.001
Mean BMI kg/m^2 ^(SD)	27.6(5.0)	28.5(4.8)	0.004	30.2(6.7)	29.1(6.4)	0.001	30.0(6.5)	29.0(6.3)	0.005
% Knee injury	15.9	18.4	0.297	15.7	15.6	0.970	16.4	16.3	0.941

Occupation characteristics									
% Physically demanding occupation^a^	83.3	43.0	<0.001	82.8	38.0	<0.001	82.2	37.4	<0.001
Mean occupational physical activities score (SD)^b^	10.7(2.6)	10.0(2.7)	<0.001	8.7(3.1)	8.8(3.0)	0.505	8.6(3.0)	8.7(3.1)	0.630

HRT use characteristics									
% Current HRT use	N/A	N/A	N/A	N/A	N/A	N/A	13.9	30.8	<0.001

Radiographic OA									
% Radiographic knee OA	32.8	22.9	<0.001	41.9	23.9	<0.001	41.9	24.9	<0.001
% Bilateral radiographic knee OA	19.9	12.1	0.001	28.7	12.7	<0.001	29.2	13.7	<0.001

Symptomatic OA									
% Symptomatic knee OA	20.2	11.1	<0.001	27.0	12.1	<0.001	27.1	13.5	<0.001
% Symptomatic bilateral knee OA	5.9	4.0	0.189	13.4	4.7	<0.001	13.1	5.0	<0.001

*P *values are listed for comparisons between <12 years and 12+ years education within each gender category (men, women, postmenopausal women).

Low educational attainment, <12 years; high educational attainment, ≥ 12 years.

BMI, body mass index; HRT, hormone replacement therapy.

^a^Laborers, fabricators, service, production, forestry, farming (as opposed to managerial, professional, technical, sales, administrative).

^b^Scale (0-16) increasing for frequency of lifting, standing, walking, and squatting.

For men, results of multivariable modeling indicate that the associations of low educational attainment with radiographic knee OA outcomes were explained by age and race, but the association with symptomatic knee OA persisted (Table 2). Men with low educational attainment have 86% higher odds of having symptomatic knee OA than do men with higher educational attainment, after additional adjustment for BMI, knee injury, current smoking, current alcohol use, and occupational factors.

**Table 2:** Odds ratios [95% CI] for educational attainment associated with four knee OA outcomes

	Men	Women	Postmenopausal women
Radiographic knee OA	n = 1,053	n = 1,574	n = 1,330
Unadjusted	1.65 [1.25, 2.17]	2.30 [1.84, 2.86]	2.18 [1.72, 2.76]
Adjusted age/race	1.12 [0.83, 1.53]	1.61 [1.26, 2.05]	1.55 [1.19, 2.01]
+ Lifestyle, clinical	1.23 [0.89, 1.70]	1.39 [1.08, 1.80]	1.35 [1.02, 1.78]
+ Occupation	1.21 [0.85, 1.71]	1.48 [1.12, 1.96]	1.34 [0.99, 1.81]
+ HRT	N/A	N/A	1.33 [0.98, 1.79]

Bilateral radiographic knee OA	n = 908	n = 1,338	n = 1,132
Unadjusted	1.81 [1.25, 2.61]	2.77 [2.09, 3.67]	2.59 [1.92, 3.49]
Adjusted age/race	1.05 [0.69, 1.59]	1.72 [1.26, 2.35]	1.68 [1.21, 2.34]
+ Lifestyle/clinical	1.17 [0.76, 1.80]	1.45 [1.03, 2.04]	1.44 [1.01, 2.07]
+ Occupation	1.06 [0.67, 1.68]	1.62 [1.12, 2.36]	1.55 [1.05, 2.30]
+ HRT	N/A	N/A	1.53 [1.03, 2.27]

Symptomatic knee OA	n = 1,052	n = 1,571	n = 1,327
Unadjusted	2.03 [1.43, 2.86]	2.69 [2.06, 3.5]	2.39 [1.81, 3.16]
Adjusted age/ethnicity	1.55 [1.06, 2.26]	1.90 [1.42, 2.54]	1.74 [1.28, 2.36]
+ Lifestyle, clinical	1.71 [1.14, 2.56]	1.66 [1.21, 2.27]	1.51 [1.08, 2.11]
+ Occupation	1.86 [1.20, 2.87]	1.64 [1.16, 2.31]	1.46 [1.02, 2.10]
+ HRT	N/A	N/A	1.46 [1.01, 2.09]

Bilateral symptomatic knee OA	n = 943	n = 1,403	n = 1,172
Unadjusted	1.50 [0.82, 2.76]	3.11 [2.08, 4.64]	2.86 [1.86, 4.41]
Adjusted age/ethnicity	0.94 [0.48, 1.84]	2.07 [1.33, 3.20]	1.99 [1.25, 3.19]
+ Lifestyle, clinical	1.07 [0.53, 2.13]	1.75 [1.10, 2.81]	1.76 [1.07, 2.91]
+ Occupation	0.90 [0.43, 1.88]	1.87 [1.13, 3.11]	1.87 [1.09, 3.20]
+ HRT	N/A	N/A	1.84 [1.07, 3.16]

Odds ratios compare low (<12 years) with high (≥ 12 years) educational attainment for having a knee OA outcome. For all models, data are restricted to individuals having a complete set of nonmissing values for their explanatory variables, and covariates are added (+) cumulatively.

Adjusted age/race, adjusted for age and race.

+Lifestyle/Clinical, additionally adjusted for alcohol and tobacco use, BMI, and knee injury.

+Occupation, additionally adjusted for occupational category, physical activity score.

+HRT, additionally adjusted for use of hormone replacement therapy.

For women, the results of multivariable modeling showed that the association of low educational attainment with all four knee OA outcomes persisted after adjustments for all covariates (Table 2). Compared with women with higher educational attainment, women with low educational attainment had about 50% to 85% higher odds of having the knee OA outcomes (Table 2).

To form the subset of postmenopausal females, about 240 women were removed (average age of 50 years and 23% low educational attainment). Overall, the results from the subset tend to show smaller odds ratios (ORs) for education than those seen from the set of all females (Table 2), but associations with bilateral radiographic OA and unilateral and bilateral symptomatic OA remain significant. The OR for education with radiographic knee OA narrowly misses significance (*P *= 0.066). However, adjustment by the occupation variables actually results in nonsignificance (*P *= 0.054), and adjustment by HRT use effects only a minor additional change. In general, the findings for the subset of postmenopausal women were similar to those for all women, and adjustment for HRT did not have any substantial influence on the results.

The ORs for education presented in Table 2 are calculated from a series of models ranging from unadjusted to fully adjusted by age, race, lifestyle, clinical, and occupation factors, and HRT for postmenopausal women. The ORs for all the independent variables from the fully adjusted models are displayed in Table 3 and include the ORs for education appearing in Table 2. Among all the covariates, age, knee injury, and BMI were significant in all models. Smoking was protective for men in radiographic OA and bilateral radiographic OA (but not for women). Alcohol was protective for women in OA and symptomatic OA (but not for men). Race did not appear to be an important factor. Occupation variables were never significant, with *P *values > 0.2 in all models. Finally, HRT was protective for postmenopausal women in knee OA (OR = 0.58) and bilateral knee OA (OR = 0.44).

**Table 3:** Odds ratios [95%CI] for all independent variables associated with four knee OA outcomes

	Men	Women	Postmenopausal women
Radiographic knee OA	n = 1,053	n = 1,574	n = 1,330
Education (low)	1.21 [0.85, 1.71]	1.48 [1.12, 1.96]	1.33 [0.98, 1.79]
Physical activity (high)	1.04 [0.76, 1.42]	1.03 [0.80, 1.31]	0.99 [0.76, 1.30]
Occupation, PD	1.04 [0.74, 1.47]	0.86 [0.65, 1.14]	0.99 [0.73, 1.34]
Age 55-64	2.30 [1.51, 3.51]	1.46 [1.06, 2.02]	1.46 [1.00, 2.12]
Age 65-74	4.39 [2.87, 6.72]	2.71 [1.92, 3.84]	2.48 [1.66, 3.72]
Age 75 and over	5.48 [3.12, 9.65]	7.13 [4.67, 10.9]	6.26 [3.87, 10.1]
African American	1.31 [0.92, 1.85]	0.92 [0.71, 1.20]	0.79 [0.59, 1.04]
Knee injury	2.82 [1.97, 4.05]	1.69 [1.24, 2.31]	1.74 [1.25, 2.42]
BMI/5	1.35 [1.16, 1.58]	1.71 [1.55, 1.89]	1.68 [1.51, 1.87]
Current smoking	0.60 [0.41, 0.89]	0.91 [0.63, 1.31]	0.87 [0.58, 1.29]
Current alcohol	1.11 [0.79, 1.57]	0.62 [0.41, 0.95]	0.66 [0.42, 1.05]
Current HRT	N/A	N/A	0.58 [0.41, 0.83]

Bilateral radiographic knee OA	n = 908	n = 1,338	n = 1,132
Education (low)	1.06 [0.67, 1.68]	1.62 [1.12, 2.36]	1.53 [1.03, 2.27]
Physical activity (high)	0.86 [0.57, 1.30]	1.02 [0.73, 1.41]	0.99 [0.70, 1.40]
Occupation, PD	1.40 [0.87, 2.25]	0.75 [0.52, 1.10]	0.81 [0.55, 1.21]
Age 55-64	4.48 [2.35, 8.55]	2.04 [1.28, 3.26]	1.87 [1.09, 3.22]
Age 65-74	8.38 [4.38, 16.0]	4.60 [2.81, 7.55]	3.92 [2.21, 6.94]
Age 75 and over	10.6 [4.68, 23.9]	13.6 [7.64, 24.2]	10.7 [5.60, 20.6]
African American	1.81 [1.16, 2.83]	1.08 [0.77, 1.52]	0.91 [0.63, 1.30]
Knee injury	2.42 [1.49, 3.94]	1.71 [1.15, 2.55]	1.62 [1.06, 2.48]
BMI/5	1.46 [1.17, 1.82]	2.08 [1.82, 2.37]	2.03 [1.76, 2.35]
Current smoking	0.59 [0.35, 0.99]	0.79 [0.46, 1.35]	0.69 [0.38, 1.24]
Current alcohol	1.20 [0.76, 1.90]	0.76 [0.43, 1.34]	0.93 [0.52, 1.68]
Current HRT	N/A	N/A	0.44 [0.26, 0.74]

Symptomatic knee OA	n = 1,052	n = 1,571	n = 1,327
Education (low)	1.86 [1.20, 2.87]	1.64 [1.16, 2.31]	1.46 [1.01, 2.09]
Physical activity (high)	0.83 [0.57, 1.22]	1.23 [0.90, 1.66]	1.22 [0.88, 1.68]
Occupation, PD	0.86 [0.56, 1.33]	1.01 [0.72, 1.44]	1.06 [0.74, 1.53]
Age 55-64	2.45 [1.40, 4.27]	2.36 [1.53, 3.64]	2.14 [1.30, 3.52]
Age 65-74	4.33 [2.49, 7.55]	3.28 [2.05, 5.26]	2.95 [1.72, 5.06]
Age 75 and over	4.83 [2.35, 9.92]	12.2 [7.18, 20.8]	10.9 [5.94, 19.9]
African American	1.13 [0.73, 1.75]	0.81 [0.59, 1.12]	0.70 [0.49, 0.98]
Knee injury	4.05 [2.68, 6.11]	2.77 [1.96, 3.92]	2.63 [1.82, 3.78]
BMI/5	1.57 [1.30, 1.90]	1.90 [1.69, 2.14]	1.88 [1.66, 2.14]
Current smoking	0.86 [0.54, 1.37]	1.22 [0.76, 1.96]	1.12 [0.68, 1.86]
Current alcohol	1.40 [0.92, 2.14]	0.52 [0.29, 0.94]	0.57 [0.31, 1.07]
Current HRT	N/A	N/A	0.75 [0.48, 1.17]

Bilateral symptomatic knee OA	n = 943	n = 1,403	n = 1,172
Education (low)	0.90 [0.43, 1.88]	1.87 [1.13, 3.11]	1.84 [1.07, 3.16]
Physical activity (high)	0.81 [0.42, 1.60]	0.96 [0.61, 1.51]	0.97 [0.60, 1.57]
Occupation, PD	1.87 [0.86, 4.06]	0.84 [0.50, 1.39]	0.85 [0.49, 1.45]
Age 55-64	5.40 [1.68, 17.4]	3.40 [1.69, 6.85]	3.99 [1.64, 9.74]
Age 65-74	9.64 [3.03, 30.7]	5.10 [2.41, 10.8]	4.92 [1.91, 12.7]
Age 75 and over	16.9 [4.42, 64.5]	18.2 [7.92, 41.9]	16.8 [6.04, 46.6]
African American	1.13 [0.54, 2.37]	0.89 [0.56, 1.42]	0.81 [0.49, 1.33]
Knee Injury	2.19 [1.03, 4.65]	2.11 [1.26, 3.52]	1.79 [1.03, 3.12]
BMI/5	1.81 [1.31, 2.50]	2.20 [1.86, 2.60]	2.12 [1.76, 2.54]
Current smoking	0.77 [0.33, 1.77]	1.33 [0.65, 2.72]	1.23 [0.57, 2.68]
Current alcohol	1.51 [0.73, 3.14]	0.62 [0.26, 1.48]	0.78 [0.32, 1.89]
Current HRT	N/A	N/A	0.48 [0.22, 1.06]

Results are taken from the fully adjusted models as presented for education in Table 2

PD, Physically Demanding (Occupation); Low educational attainment, <12 years; Occupationally related Physical Activity Score, high is ≥ 10 on a scale of 0-16 for lifting, standing, walking, and squatting.

BMI/5 is the continuous, model variable; the OR represents an increase of 5 units of BMI.

The referent class for age is 45-54 years.

In exploratory models with all potential covariates, run separately by gender, no interaction terms for educational level with age group or race were found to be statistically significant at the 0.10 level, by using the Bonferroni correction for multiple tests, and so no further stratification was necessary.

Finally, as a check on the effect of missing values, particularly the Census occupation code, characteristics prepared from the original sample of 3,047 participants showed no notable differences from those presented in Table 1. A modeling analysis was repeated but with removal of the grouping of occupational variables, leaving a sample of 2,914. Results were similar in OR magnitudes to those of Table 2, and the findings of significance were unchanged, except, in the case of postmenopausal women adjusted for HRT, formal significance of the OR for education associated with knee OA was restored (*P *= 0.034).

## Discussion

This is the first study to examine the effect of demographic, lifestyle, clinical, and occupational factors on the relation between educational attainment and radiographic, bilateral radiographic, symptomatic, and bilateral symptomatic knee OA in a rural, biracial setting. Our results confirm that those with limited educational attainment are more likely than are those with higher educational attainment to have these OA outcomes in unadjusted analyses. This is consistent with evidence demonstrating associations between educational attainment and outcomes in RA [7,8,10-14]. As was noted in discussions regarding the associations of educational attainment with RA outcomes [39,40], the effects of educational attainment in OA outcomes are most likely not direct but rather indirect through other factors (for example, demographic, lifestyle, clinical, and occupational factors). Because educational attainment is unlikely to be amenable to intervention in adulthood, it is critical to identify factors that might explain these observations to target potentially modifiable risk factors accounting for the education-related disparities in knee OA outcomes.

In the early twentieth century in the rural South, it was not uncommon for individuals to leave school before completion of a high school degree (Wilma Howard, personal communication), and our study reflects this fact. Older individuals in our study were much less likely than were those in younger age groups to have completed high school. In men, age and race explained the associations between educational attainment and radiographic and bilateral radiographic knee OA. In women, associations between educational attainment and radiographic and bilateral radiographic knee OA were only partially explained by age, race, BMI, knee injury, current smoking, or current alcohol use. Current HRT use has been shown to be inversely associated with radiographic knee OA [26,32,33], and is less likely to be used by those in low SES groups [26-31]. In our study, the education effect on radiographic or bilateral radiographic knee OA in postmenopausal women was not explained by current HRT use.

Occupational physical demands, such as knee bending and heavy lifting, have been identified as risk factors for radiographic knee OA [41-45] and can vary with educational attainment. Indeed, Hannan and colleagues [16] included occupation (among other factors, including age, race, obesity, and knee injury, but not smoking, alcohol use, or HRT) in their analysis of education and radiographic knee OA, and noted that the association between education and radiographic knee OA could be explained by these factors. Their analysis did not examine occupational physical activity beyond knee bending, and it was limited to individuals aged 35 to 64 years who were not homemakers. In addition, they did not look at men and women separately nor did they examine postmenopausal women specifically. These differences in study design, analysis, and assessment of covariates may explain the differences between our study and theirs.

An additional factor that may explain associations between educational attainment and radiographic knee OA outcomes in women is diet [46]. Several dietary factors, including vitamins C and D, have been identified as possible protective factors for radiographic knee OA progression [47]. Dietary intake of these vitamins may vary by SES [48], and lack of adequate exposure to these potentially protective factors could possibly explain the association between educational attainment and radiographic knee OA. However, we did not have data on dietary intakes, rendering assessment of these factors beyond the scope of this analysis.

Similar to the results of Hannan and colleagues regarding educational attainment and symptomatic knee OA, we found that, in both men and women, associations between limited educational attainment and symptomatic knee OA could not be fully explained by the factors we evaluated. Psychological variables, such as depression, self-efficacy, or learned helplessness, have been associated with joint symptoms [49] and might help to explain these associations.

Our study has some limitations, including the fact that this is a cross-sectional analysis. However, because final educational level is usually attained before adulthood and certainly before the age at which OA usually develops, it is unlikely that many, if any, individuals developed OA before they completed their education. We did not include assessment of the patellofemoral joint in our analysis, and this may have caused us to misclassify some individuals with isolated patellofemoral OA as unaffected. This might have diluted the strength of our findings but is unlikely to have caused inflated estimates or associations that were spurious.

We do not have information on treatment history, and individuals with lower educational attainment may be less likely to seek medical help or could have poorer access to health care.

## Conclusions

In conclusion, those with limited educational attainment were more likely than those with higher educational attainment to have radiographic knee OA, bilateral radiographic knee OA, symptomatic knee OA, and bilateral symptomatic knee OA. In men, associations between education and radiographic knee OA outcomes did not remain significant after adjustment by age/race, whereas in women, demographic, lifestyle and clinical and occupational factors did not totally explain these associations. Symptomatic knee OA was also more common in both men and women with limited educational attainment, and these observations also remained unexplained by these risk factors. Given the growing public health impact of OA in the United States today, and its strong association with limited educational attainment, further research is needed to identify factors, such as diet and environmental exposure, that might be amenable to intervention to prevent radiographic and symptomatic knee OA.

## Abbreviations

BMI: body mass index; HRT: hormone replacement therapy; K/L grade: Kellgren-Lawrence grade; OA: knee osteoarthritis; PD: physically demanding occupation; RA: rheumatoid arthritis; SES: socioeconomic status.

## Competing interests

The authors declare that they have no competing interests.

## Authors' contributions

LFC, JS, and BCS contributed to study conception and design, participated in data analysis and interpretation, and in the drafting and reviewing of the manuscript. JC, TS, JR, BS, CH, and JMJ contributed to study design and interpretation and reviewing of the manuscript. All authors read and approved the final manuscript.
